# Patient Outcomes After Penile Prosthesis Placement with Concomitant Non-Reconstructive Urologic Procedures

**DOI:** 10.1590/S1677-5538.IBJU.2024.0332

**Published:** 2025-01-10

**Authors:** Ridwan Alam, William S. Du Comb, Jason A. Levy, Arthur L. Burnett

**Affiliations:** 1 Johns Hopkins University School of Medicine Department of Urology The James Buchanan Brady Urological Institute Baltimore MD USA The James Buchanan Brady Urological Institute, Department of Urology, Johns Hopkins University School of Medicine, Baltimore, MD, USA

**Keywords:** Erectile Dysfunction, Penile Prosthesis, Urologic Surgical Procedures, Male

## Abstract

**Purpose:**

There is substantial literature demonstrating minimal to no increased risk of three-piece penile prosthesis (PP) complications for patients undergoing placement with concomitant reconstructive urologic procedures. However, there is a paucity of research investigating outcomes for patients suffering from erectile dysfunction (ED) who undergo concomitant non-reconstructive urologic procedures at the time of PP placement.

**Materials and Methods:**

We performed a retrospective review of patients undergoing PP placement and a second non-reconstructive urologic procedure performed concomitantly at our institution between January 2007 and July 2021. This was compared to a control cohort of 127 patients who underwent PP placement only. Outcomes of interest were complications and device infections. Comparative statistics were used to compare the two groups, and the Kaplan-Meier method was used to estimate the rate of complications and infections over time.

**Results:**

We identified 44 patients who underwent concomitant surgery and 127 patients who underwent single surgery only. The types of concomitant surgeries were as follows: 23 endoscopic (52.3%), 9 penile (20.5%), 10 scrotal (22.7%), 1 hardware placement (2.3%), and 1 oncologic (2.3%). Hypertension was the only comorbidity that was more prevalent in the concomitant group (65.9% vs. 43.8%, P<0.01). Patients undergoing concomitant surgery had similar complication (4.6% vs. 3.6%, P=0.79) and device infection (2.3% vs. 0.7%, P=0.43) rates as the single surgery group.

**Conclusions:**

In the largest study of its kind, we observed that patients undergoing concomitant non-reconstructive urologic procedures at the time of PP placement are not at an increased risk of adverse events.

## INTRODUCTION

Erectile dysfunction (ED) is the inability to attain or maintain an erection firm enough for satisfactory sexual intercourse (1). Treatment options for ED include lifestyle modifications, oral therapy, vacuum pumps, intraurethral suppositories, intracavernosal injections, and surgery (2). Per the American Urological Association (AUA) guidelines, men may be offered all treatment options upfront with a clear understanding of the risks and benefits of each (1). However, penile prosthesis (PP) implantation remains the gold standard for patients who cannot tolerate or fail less invasive treatment options, as well as for those who are not candidates for such options.

Conventionally, PP implantation is performed as a standalone procedure. This dogma was founded on the tenet that concomitant procedures would increase operative time, local wound exposure, and post-operative edema – factors which can potentially increase infection risk and loss of the prosthesis. Thus, caution has been advised against concomitant procedures due to the presumed increased risk of bacterial seeding (3).

This tenet has been challenged in multiple series which demonstrated no increased risk of adverse events. In fact, potential advantages of concomitant reconstructive procedures were touted. For example, with concomitant PP and artificial urinary sphincter (AUS) or male sling implantation, patients returned to sexual activity and regained urinary continence faster than when these surgeries were performed independently (46). Furthermore, penile straightening procedures associated with Peyronie's disease were commonly performed with implantation of a PP, with high patient satisfaction rates and low risk of adverse events (7). Procedures such as suprapubic lipectomy, ventral phalloplasty, or suspensory ligament release can be carried out at the time of penile implant surgery with no increased risk of complications (8). While these observations are encouraging for the prospect of concomitant surgeries during device implantation, only reconstructive urologic procedures performed at the time of PP placement have been studied.

As such, there is a need to examine the outcomes of patients undergoing non-reconstructive urologic procedures at the time of PP placement. In this study, we sought to examine the long-term outcomes of patients undergoing PP placement with a concomitant non-reconstructive urologic procedure.

## MATERIALS AND METHODS

This study was approved by the Johns Hopkins University Institutional Review Board (IRB00205900). A retrospective review of patients undergoing PP placement at Johns Hopkins between January 2007 to July 2021 was conducted. Our institutional records were queried for patients who underwent PP implantation along with any other procedure using Current Procedural Terminology (CPT) codes. Any patient who underwent adjunctive penile reconstructive procedures (e.g., penile modeling, penile plaque excision) or anti-incontinence surgeries were excluded. Additionally, patients with neurogenic bladders were excluded from the study because previous studies have documented an increased risk of complications after PP implantation in this patient population (9).

The control group consisted of patients who underwent PP implantation only between July 2016 and July 2021. It was not necessary to increase the time range for the control group as the ratio was 3:1 to the concomitant group, which was statistically adequate. These patients underwent first-time implants due to organic vasculogenic disease, a history of radical prostatectomy, or a history of pelvic radiation. Exclusion criteria for the control group was a history of priapism and prior gender-affirming surgery due to inherently increased risks of complications associated with these patients (10).

Patient records were examined for baseline patient characteristics, operative details, and follow-up information. Any complications attributable to the surgery were recorded for the entire duration of follow-up.

Comparative statistics (Mann-Whitney U test, χ^2^ test, Fisher's exact test) were used to compare characteristics between the two groups. The time to postoperative complications was estimated using the Kaplan-Meier method. All analyses were performed using Stata 17.0, and statistical significance was set at α=0.05.

## RESULTS

We identified 44 patients who underwent concomitant procedures and 137 patients who underwent PP implantation only. There were no significant differences in age, body mass index (BMI), total number of comorbidities, marital status, or smoking status between groups (Table-1). There was a significantly higher proportion of white patients undergoing concomitant surgery compared to single surgery (68.2% vs. 41.6%, P=0.007). Furthermore, while there were no differences in the rate of cardiovascular disease or diabetes mellitus between the two groups, there was a significantly higher proportion of hypertensive patients in the concomitant surgery group (65.9% vs. 43.8%, P=0.01).

**Table 1:** Baseline characteristics of patients undergoing concomitant surgery and single surgery.

Characteristic		Concomitant Surgery (n=44)	Single Surgery (n=137)	P-value
Median age at surgery, years (IQR)		63.8 (53.7-68.4)	65.0 (57.8-69.1)	0.16
**Race, n** (%)				0.007
	White	30 (68.2%)	57 (41.6%)	
	Black	13 (29.6%)	68 (49.6%)	
	Other	1 (2.3%)	12 (8.8%)	
Median BMI, kg/m^2^ (IQR)		31.2 (27.1-32.3)	28.1 (26.0-32.9)	0.30
Married, *n* (%)		29 (65.9%)	84 (61.3%)	0.58
**Comorbidities, *n*** (%)				
	Cardiovascular disease	12 (27.3%)	23 (16.8%)	0.13
	Diabetes mellitus	13 (29.6%)	53 (38.7%)	0.27
	Hypertension	29 (65.9%)	60 (43.8%)	0.01
**Number of comorbidities, n** (%)				0.17
	0	9 (20.5%)	35 (25.6%)	
	1	20 (45.5%)	71 (51.8%)	
	2	11 (25.0%)	28 (20.4%)	
	3	4 (9.1%)	3 (2.2%)	
Smoker, n (%)		19 (43.2%)	56 (40.9%)	0.79

Almost 90% of patients received an American Medical Systems (AMS) device (Boston Scientific, Marlborough, MA), with the remaining 10% receiving a Coloplast device (Coloplast A/S, Humlebaek, Denmark). No difference was seen between the concomitant or single surgery groups with respect to the device used (P=0.83). All patients in both cohorts underwent PP implantation via a penoscrotal approach. No cases were identified in which a patient had a two-piece or malleable PP and a concomitant procedure.

Twenty-two patients undergoing concomitant surgery had a cystoscopic procedure (50.0%) (Table-2). Scrotal surgeries, including vasectomy, orchiectomy, hydrocelectomy, and spermatocelectomy, comprised 10 cases (22.7%). Penile surgeries, including circumcision and release of glandular adhesions, were performed in 9 cases (20.5%). Ureteroscopy with laser lithotripsy, sacral neuromodulator device implantation, and radical retropubic prostatectomy were performed in 1 patient each (2.3% each).

**Table 2:** Operative and follow-up details of patients undergoing concomitant surgery and single surgery.

Detail			Concomitant Surgery (n=44)	Single Surgery (n=137)	P-value
**Type of penile prosthesis, n (%)**					0.83
	AMS		39 (88.6%)	123 (89.8%)	
	Coloplast		5 (11.4%)	14 (10.2%)	
**Concomitant procedure type, n (%)**					
	Cystoscopy		22 (50.0%)		
	Scrotal surgery		10 (22.7%)		
		Vasectomy	5		
		Orchiectomy	3		
		Hydrocelectomy	1		
		Spermatocelectomy	1		
	Penile surgery		9 (20.5%)		
		Circumcision	8		
		Lysis of adhesions	1		
	Lithotripsy		1 (2.3%)		
	Hardware		1 (2.3%)		
	Radical prostatectomy		1 (2.3%)		
Follow-up time, months (IQR)			7.4 (1.6-27.9)	4.9 (2.8-9.2)	0.28
Postoperative complication, n (%)			2 (4.6%)	5 (3.6%)	0.79
Device infection, n (%)			1 (2.3%)	1 (0.7%)	0.43

The median follow-up time was 7.7 months for the concomitant surgery group and 4.9 months for the single surgery group (P=0.22) (Table-2). There was no significant difference in complications between the two groups (4.6% concomitant vs. 3.6% single, P=0.79). The device infection rate was comparable as well (2.3% concomitant vs. 0.7% single, P=0.43).

**Figure 1:**
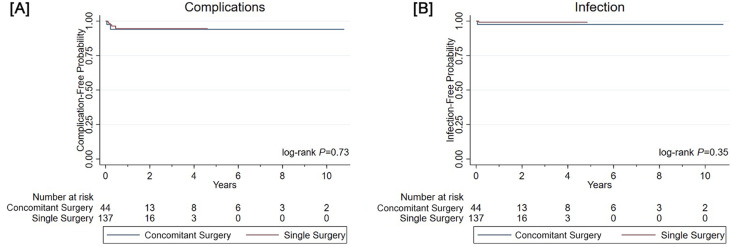
Kaplan-Meier curves comparing concomitant surgery and single surgery for (A) freedom from complications and (B) freedom from device infections.

Patients who underwent concomitant surgery had the following complications (n=2): device infection at one month (release of glandular adhesions) and device erosion at two months (circumcision). Patients who underwent PP surgery only had the following complications (n=5): device infection at one month; pump failure at one month; fluid leak at three months; wound separation at six months; pump failure at six months. There was no difference in the time complication between the two groups (P=0.73) (Figure-1A). The rates of freedom from complication at 3, 6, and 12 months was 94.0% throughout for the concomitant surgery group and 97.4%, 94.6%, and 94.6%, respectively, for the single surgery group. Similarly, there was no difference in the time to infection (P=0.35) (Figure-1B). The rate of freedom from infection was 97.6% for the concomitant surgery group and 99.2% for the control group at 3, 6, and 12 months.

## DISCUSSION

In recent years, the dogma that PP implantation should not be performed in conjunction with other procedures has been challenged. However, it is unknown whether non-reconstructive urologic procedures can be performed safely without compromising outcomes. To this end, we examined patients undergoing PP implantation and concomitant non-reconstructive urologic procedures over a 14- year span at a high-volume institution and found no increased risk of postoperative complications or device infections.

Concomitant surgeries with PP implantation have been increasingly performed over the past decade, the majority of which are reconstructive. Examples of reconstructive procedures include the correction of Peyronie's disease, stress urinary incontinence, and penile length (4-8,11,12). These studies have shown that reconstructive urologic procedures performed concurrently with PP implantation confer no increased risk of adverse outcomes. However, there are limited studies evaluating concomitant non-reconstructive urologic procedures at the time of PP implantation.

Case reports have been published demonstrating the feasibility of non-reconstructive urologic procedures at the time of PP placement, and early evaluation suggests that patient-reported quality of life is improved with concomitant surgery without compromising surgical outcomes (13-15). However, these case reports were very limited in sample size. To our knowledge, the present study is the largest to date rigorously examining outcomes in this patient population. Importantly, we found that complication rates in patients undergoing concomitant procedures were similar to those of individuals undergoing first-time implantation.

Furthermore, we found that when complications arise, they tend to do so within the first three months, and of those complications, infection rates between the concomitant surgeries and PP implantation alone were comparable. Overall infection rates for the concomitant surgery group resemble those in the literature for implantation of a PP alone (1% to 3%) (16,17). These data offer additional support for performing concomitant procedures. Notably, most of the patients in both groups opted for an AMS device as opposed to a Coloplast device. At our institution, we provide patients with both options and review the pros and cons of each device prior to selection.

Apparent benefits of concomitant procedures include one setting for surgical intervention, obviating the need for further induction of anesthesia during subsequent procedures. Financial savings may also be potentially realized by utilizing the same operating room equipment and staff. Conversely, depending on the institutional setting, reimbursement for concomitant procedures may be reduced for the surgeon performing the operation (18). Thus, while concomitant procedures appear feasible and safe, the relative paucity of data may be driven in part by non-medical processes which disincentivize these types of procedures.

Insertion of a PP remains a common procedure for patients with ED and is performed by both fellowship-trained urologists and general urologists. While a urologist who is a high-volume implanter may feel comfortable performing concomitant procedures at the time of PP implantation, a general urologist may be apprehensive. Our report may seem more applicable for high-volume implanters, and such surgeons may have performed concomitant surgeries occasionally in the past with a modest sense of their routineness. Our report serves to affirm the success of concomitant procedures with PP implantation.

There are several limitations which should be noted. The number of patients undergoing urologic procedures at the same time as PP implantation in our series is relatively low at 44 patients. However, we present the largest examination of this patient population to date, and with a dataset spanning over 14 years, the low numbers suggest the infrequency with which these concomitant procedures are performed. Furthermore, the outcomes we observed are thankfully relatively rare, which limits the amount of analysis that can be soundly performed (i.e., multivariable regression). Nevertheless, even without adjusting for baseline patient characteristics, which were not significantly different between the two groups, we found no difference in both short- and long-term postoperative complications and infections. Finally, our sample consisted of patients undergoing surgery at a high-volume tertiary care center and may not be generalizable to the broader community.

In this study of patients undergoing PP implantation with a concomitant non-reconstructive urologic procedure, we find no increased risk of complications or device infections when compared to patients undergoing first-time PP placement only. While further investigation is needed, our findings challenge the traditional dogma that secondary urologic procedures should be avoided at the time of PP implantation.
